# Comparative chemical profiling of leaf essential oils from *Cinnamomum kanehirae* and related species using steam distillation and solvent extraction: Implications for plant-based classification

**DOI:** 10.1016/j.heliyon.2024.e30628

**Published:** 2024-05-03

**Authors:** Wen-Hui Chen, Ya-Zhu Ko, Hsiu-Chun Chang, Chui-Shiang Chang, Kuo-Hsiang Hung, Huie-Chuan Shih, Li-Ping Ju, Meng-Shin Shiao, Yu-Chung Chiang

**Affiliations:** a Department of Biological Sciences, National Sun Yat-sen University, Kaohsiung, 804, Taiwan; b Pingtung County Central Laboratory, No.272, Je-Yu Road, Pingtung, 900, Taiwan; c Graduate Institute of Bioresources, Pingtung University of Science and Technology, Pintung, 912, Taiwan; d Department of Nursing, Meiho University, Pingtung, 912, Taiwan; e Forest Rrotection Division, Taiwan Forestry Research Institute, Taipei, 100, Taiwan; f Research Center, Faculty of Medicine Ramathibodi Hospital, Mahidol University, Bangkok, 10400, Thailand; g Department of Biomedical Science and Environment Biology, Kaohsiung Medical University, Kaohsiung, 807, Taiwan; h The Multidisciplinary and Data Science Research Center(MDSRC), National Sun Yat-sen University, Kaohsiung, 804, Taiwan

**Keywords:** *Cinnamomum kanehirae*, *Cinnamomum micranthum*, *Cinnamomum camphora*, Essential oil, Gas chromatography/mass spectrometry (GC/MS), Chemotype

## Abstract

*Cinnamomum kanehirae Hayata,* belonging to Lauraceae family, is an indigenous and endangered species of considerable economic importance in Taiwan. It plays a crucial role as the host for the economically valuable saprotrophic fungus, *Taiwanofungus camphorates*. However, accurate species identification poses a challenge due to the similarity in morphological features and frequent natural hybridization with closely related species. Acquiring high-quality and pure leaf oils becomes imperative for precise species identification and producing superior goods. In this study, our objective was to establish methodologies for analyzing the chemical composition of leaf essential oils and subsequently apply this knowledge to differentiate among three *Cinnamomum* species. Gas chromatography-mass spectrometry (GC/MS) was employed to scrutinize the chemical makeup of leaf essential oils from three closely related species: *C. kanehirae*, *C. micranthum*, and *C. camphora*. We utilized Steam Distillation (SD) and steam distillation-solvent extraction (SDSE) methods, with the SDSE-Hexane approach chosen for optimization, enhancing extraction efficiency and ensuring essential oil purity. Through the SDSE-Hexane method, we identified seventy-four compounds distributed across three major classes: monoterpenes hydrocarbons (0.0–7.0 %), oxygenated monoterpenes (3.8–90.9 %), sesquiterpenes hydrocarbons (0.0–28.3 %), and oxygenated sesquiterpenes (1.6–88.1 %). Our findings indicated the presence of more than one chemotype in both *C. kanehirae* and *C. camphora*, whereas no specific chemotype could be discerned in *C. micranthum*. Furthermore, clustering based on chemotypes allowed for the differentiation of samples from the three species. Notably, we demonstrated that the chemical compositions of grafted *C. kanehirae* remained largely unaffected by the rootstock. Conversely, natural hybrids between *C. kanehirae* and *C. camphora* exhibited profiles more closely aligned with *C. kanehirae*. The optimized extraction method and the chemotype-based classification system established in this study present valuable tools for essential oil preparation, species identification, and further exploration into the genetic variation of *Cinnamomum*.

## 1 Introduction

The genus *Cinnamomum* Schaeff. comprises approximately 250 species, predominantly found in tropical and subtropical regions across Asia, Pacific islands, and Australia [1,2]. *Cinnamomum kanehirae* Hayata, an autochthonous species exclusive to Taiwan, thrives in broad-leaved forest ecosystems situated at altitudes ranging from 200 to 2000 m [[3], [4], [5]]. *Cinnamomum kanehirae* is economically and ecologically significant in Taiwan [2,6]. Its wood and leaf extracts have anti-inflammatory, anti-oxidant, and anti-carcinogenic properties [[6], [7], [8], [9]]. Besides, its wood is used in high-quality furniture making [2]. Similarly, its related species, *C. camphora*, has multiple uses, including medicine, building and furniture, aromatics, and pesticides [[10], [11], [12], [13], [14]]. The natural population of *C. kanehirae* trees in the wild has recently declined rapidly, which is attributed to the loss of forest land and excessive logging of the trees [4,5,15]. Despite stringent regulations governing the logging of this species in Taiwan, instances of illegal logging persist.

Distinguishing itself from other *Cinnamomum* species, *C. kanehirae* serves as the exclusive host for the saprotrophic fungus *Taiwanofungus camphorates*, also known as *Antrodia cinnamomea*. This medicinal fungus holds immense market value due to its diverse biological activities, encompassing antioxidant, anti-inflammatory, anti-tumor, hepatoprotective, and immunomodulatory properties [[16], [17], [18], [19], [20], [21]]. Various cultivation methods, such as solid or liquid mediums and mixed types of sawdust, can be employed to culture *T. camphorates* for cost-effective product generation. However, the resultant compositions of beneficial medicinal compounds in fungi cultivated through these methods do not match those derived from growth on the trunk of *C. kanehirae* [7,22]. While *T. camphorates* can be cultivated on closely related species such as *C. camphora*, the resulting chemical compositions differ and lack the medicinal effects found in fungi cultivated on *C. kanehirae*. Moreover, consumption of *T. camphorates* cultivated on *C. camphora* has been associated with adverse health effects [[23], [24], [25]]. Consequently, the imperative to use *C. kanehirae* for *T. camphorates* cultivation stands as a primary driver for excessive logging, leading to a significant decline in *C. kanehirae* populations.

The identification of *C. kanehirae* poses a challenge due to morphological similarities and the occurrence of natural hybridization with closely related species, specifically *C. micranthum* and *C. camphora* [[26], [27], [28]]. Notably, relying on a section of logged wood for species identification is impractical. Furthermore, instances of natural hybridization have been documented in areas where *C. kanehirae* and *C. camphora* coexist, particularly in northern and southeastern Taiwan [28]. The presence of intermediate morphological characteristics in some *C. kanehirae* seed orchard seedlings supports the evidence of hybridization with *C. camphora*. Hybrids exhibit leaf characteristics on the backside that are an intermediary between the glossy and bright features of *C. kanehirae* and the powdery white wax-covered traits of *C. camphora* [[28], [29], [30]]. This hybridization introduces uncertainty and confusion, especially concerning the legal cultivation and subsequent economic value of *T. camphorates*.

In the first edition of the Flora of Taiwan, *C. kanehirae* and *C. micranthum* were merged into a single species, *C. micranthum* (Hay.), due to their similar morphologies [23]. However, subsequent differentiation established them as distinct species *C. kanehirae* Hay. and *C. micranthum* (Hay.) Hay. based on fruit morphology, alloenzyme data, and chloroplast genomic sequence information [3,4,22,27]. Recently, molecular evidence suggests that *C. kanehirae* and *C. micranthum* should be treated as the same species and renamed *Camphora micranth* [[32], [33], [34]]. The inconsistencies in the circumscription of closely-related *Cinnamomum* species in various studies underscore the potential utility of alternative tools, such as chemotypes in the essential oil extracted from leaves, to aid in species delimitation.

From the literature, various studies have reported different numbers of chemotypes of essential oils present in the leaves of *C. kanehirae*. One study identified five chemotypes (linalool type, linalool/1,8-cineole type, linalool/α-cadinol type, 1,8-cineole type, and mixed type) while another study identified four chemotypes (eucalyptol type, isonerolidol type, linalool type, and tetradecanal type) [2,35]. The differences in the amounts of the main compounds in essential oils extracted from the leaves may be attributed to various environmental factors in different geographic regions, which was reported in *C. camphora* [35]. Additionally, several studies have compared chemical compositions among closely related species in *Cinnamomum* taxa and showed that the compositions in the essential oils are distinguishable between *C. kanehirae* and *C. micranthum* [22,[36], [37], [38]]. This suggested the possibility of using chemotypes to distinguish the three *Cinnamomum* species in Taiwan.

In 1980, the Taiwan Forestry Bureau established a *C. kanehirae* seed orchard in Sanmin nursery, Yuli, Hualien, using 2-year-old *C. camphora* as rootstock and selecting natural *C. kanehirae* bud wood. During the 10-year cultivation period, some grafted *C. kanehirae* exhibited leaves similar to *C. camphora* (covered with white powder) in their juvenile stage but gradually reverted to typical *C. kanehirae* leaf morphology. This indicated that rootstock could affect the growth of grafted *C. kanehirae* [39]. However, the chemical compounds of the grafted trees have never been examined.

Currently, there is a lack of comprehensive research comparing the chemical compound compositions among the three *Cinnamomum* species in Taiwan. In order to gain a thorough understanding and effectively manage the economic applications of these species, this study was designed to accomplish three primary objectives. First, the primary goal of this study is to assess different extraction methods and establish an effective technique for extracting essential oil from the leaves of *C. kanehirae*, *C. micranthum*, and *C. camphora*. The central focus lies in optimizing the extraction process to maximize the yield and enhance the quality of essential oils, facilitating subsequent compound identification. Second, the study aims to identify chemotypes present in the leaf essential oils of *Cinnamomum* species. The objective is to pinpoint chemotypes of leaf essential oils of *C. kanehirae*, *C. micranthum*, and *C. camphora*, aiming to differentiate and categorize these closely related *Cinnamomum* species based on their distinctive chemical compositions. This will provide insights into the chemical diversity and variability within and among the *Cinnamomum* species. Lastly, the study investigates the influence of grafting and natural hybridization on the chemical composition of leaf essential oils in the *Cinnamomum* species. The objective is twofold: first, to scrutinize the compounds in the leaf essential oil of hybrid individuals, and second, to explore whether grafting *C. kanehirae* onto *C. camphora* rootstock impacts the original chemical composition. Anticipated outcomes of this study include providing vital information for taxonomy and species identification offering valuable insights into the chemical polymorphism of leaves.

## 2 Materials and methods

### 2.1 Plant materials

**Fig. 1:**
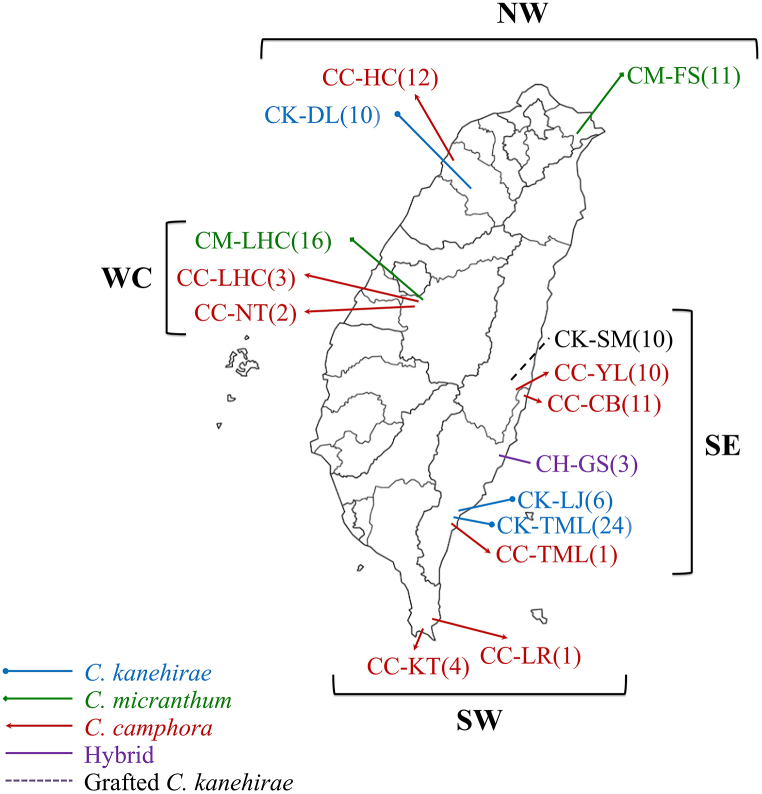
The sampling populations and regions of three *Cinnamomum* species, grafted *C. kanehirae*, and natural hybrids. The symbols of populations are listed in Table 1.

This study investigated 124 samples from three closely related *Cinnamomum* species: *C. kanehirae*, *C. micranthu*, and *C. camphor*, as well as grafted seedlings of *C. kanehirae* on *C. camphora* rootstock and natural hybrid samples. The specimens were collected from Taiwan's intermediate and low altitudes (20–1300 m). The sampling locations are detailed in Fig. 1 and Table 1.

**Table 1:** Sampling location and sample number of three *Cinnamomum* species, grafted *C. kanehirae*, and natural hybrids. SE: the southeastern region, NW: the northwestern region, WC: the west-central region, SW: the southwestern region.

Species	Region	Symbol	Locations	No. of samples
*C. kanehirae*	SE	CK-TML	TaiMaLi, Taitung	24
	SE	CK-LJ	LiJia, Taitung	6
	NW	CK-DL	DaLu, Hsinchu	10
*C. micranthum*	NW	CM-FS	FuShan, Yilan	11
	WC	CM-LHC	LianHua Pond, Nantou	16
*C. camphora*	SE	CC-LHC	LianHua Pond, Nantou	3
	SE	CC-YL	YuLi, Hualien	10
	SE	CC-CB	ChangBin, Taitung	11
	SW	CC-LR	LanRen Creek, Pingtung	1
	SW	CC-KT	KenTing, Pingtung	4
	WC	CC-TML	TaiMaLi, Taitung	1
	WC	CC-NT	NanTou City	2
	NW	CC-HC	HsinChu City	12
Hybrid	SE	CH-GS	GuanShan, Taitung	3
Grafted *C. kanehirae*	SE	CK-SM	SanMin Nursery, Hualien	10

A total of 40 samples of *C. kanehirae* were collected from three locations, including 24 from Taichung's Taimali (CK-TML), 10 from Hsinchu's Dalu Forest Road (CK-DL), and 6 from Taitung's Lijia Forest Road (CK-LJ). A total of 27 samples of *C. micranthum* were collected from two locations including 11 from Yilan's Fushan (CM-FS) and 16 from Nantou's Lianhua Pond (CM-LHC). A total of 44 samples of *C. camphora* were collected from eight locations including 1 from Taichung's Taimali (CC-TML), 10 from Hualien's Yuli (CC-YL), 11 from Taitung's Changbin (CC-CB), 1 from Pingtung's Lanren Creek (CC-LR), 4 from Pingtung's Kenting (CC-KT), 3 from Nantou's Lianhua Pond (CC-LHC), 2 from Nantou City (CC-NT), and 12 from Hsinchu City (CC-HC). Three hybrid samples were collected from Taitung's Guanshan (CH-GS). Ten grafted seedlings of *C. kanehirae* samples were taken from Hualien's Sanmin Nursery (CK-SM). All species were identified by Dr. Li-Ping Ju from the Taiwan Forestry Research Institute (TFRI). All leaf samples were air-dried and frozen at −20 °C before analysis.

Four geographic regions were defined: the northwestern region (NW) includes CC-HC, CM-FS, and CK-DL populations; the west-central region (WC) includes CM-LHC, CC-LHC, and CC-NT populations; the southwestern region (SW) includes CC-KT and CC-LR populations; and the southeastern region (SE) includes CK-SM, CC-YL, CC-CB, CH-GS, CK-LJ, CK-TML, and CC-TML populations.

### 2.2 Two different processes of essential oil extraction

Steam Distillation (SD) [2]: essential oil was extracted from 25 g of dried leaves in a Clevenger-type apparatus for 6 h.

Steam Distillation combined with Solvent Extraction (SDSE) [40]: 25 g of dried leaves were subjected to steam distillation equipped with a cooling system device (EYELA CA-111, Japan) for 6 h followed by treatment of different solvents for 6 h. Different solvents include acetone, hexane, and ethyl acetate (EA) (HPLC grade, Fluka, Germany). After shaking for 20 min, the upper layer of liquid was taken and dried with anhydrous sodium sulfate (analytical grade, Wako Pure Chemical Industries, Ltd., Japan) to remove excess water. Then solvent fraction was concentrated, and the volume was adjusted to 10 mL using a rotary vacuum concentrator (DLAB, RE100-Pro, Scientific Inc., USA).

The methods mentioned above were conducted in quintuplicate, with three samples each.

### 2.3 Gas chromatography/mass spectrometry (GC/MS) analysis

The essential oils contents of the collected samples obtained from SD and SDSE methods were analyzed using GC/MS, and different extraction methods were compared to determine the optimal one. The results showed that SDSE-Hexan was the most effective extraction method, with detailed findings in the Results and Discussion section. Following this, various distillation times (2, 3, 4, and 6 h) of SDSE-Hexan were compared and analyzed using GC-MS to identify the most efficient timing for the SDSE-Hexan extraction method. A repeatability test was conducted by quintuplicate with three samples, showing mean (M) ± standard deviation (SD). A statistical analysis was conducted to determine whether there were any significant differences in distillation times (2, 3, 4, and 6 h) of SDSE-Hexan for *C. kanehirae*, *C. micranthum*, and *C. camphora*. The aov (.) function in R version 4.3.2 software [41] was used for this purpose. If the F values were significant (*p* < 0.05), a post hoc Tukey test function TukeyHSD (.) was employed. A p-value of less than 0.01 was considered significant (*p* < 0.001). The data was visualized with error bars using the ggplot2 package [42] in R version 4.3.2 software.

The GC-MS system (7000C–7890B, Agilent Technologies Inc., Santa Rosa, CA, USA) equipped with a DB-5 capillary column (30 m in length, 0.25 mm in inner diameter, 0.35 μm in film thickness; Agilent Technologies Inc., Santa Rosa, CA, USA) and a back-flush device was employed to analyze the obtained essential oils. The sample was injected with a volume of 1uL. The injector port temperature was set to 250 °C, and the carrier gas was high-purity helium gas (99.9995 %). The gas was split into two streams with flow rates of 1.0 and 1.2 mL/min, respectively, and the split ratio was 10:1. The oven temperature was initially maintained at 50 °C for 2 min, then increased at a rate of 3 °C/min to 200 °C and held for 1 min, and then further increased at a rate of 10 °C/min to 250 °C, which was held for 2 min. The ionization source temperature was set to 230 °C and the ionization voltage was the electron impact ionization mode (70 eV). The mass scan range was 40–400 atomic mass units (amu), and the peak width was set at 20 s [2].

### 2.4 Peak identification and statistical analysis

The Total Ion Chromatogram (TIC) data acquired by GC-MS was subjected to analysis using Qualitative Analysis B.07.00 software (Agilent, USA). The characteristic compounds were identified based on the standard mass spectra from the NIST 14.0 mass spectrum database (National Institute of Science and Technology, USA). The characteristic compounds were matched against the spectra in the database to determine their identities.

Additionally, Kovats indices (KI) were employed for compound comparison. The KI of all volatile components were calculated using Saturated Alkanes (C8–C22). Kovats indices serve as a valuable tool in gas chromatography (GC) for assessing the relative retention times of compounds with respect to a reference compound under specific stationary phase conditions. By calculating the Kovats indices, unknown compounds can be compared to known compounds with established indices to determine their identities. To use Kovats indices for compound matching, the retention time (tR) of the unknown compound is compared to the retention times of a series of n-Alkanes with known chain lengths (tR(N) and tR (N+1)). The retention time of the unknown compound (tR(x)) should fall between the retention times of the two adjacent n-Alkanes. The Kovats index (KI) is then calculated using the formula [2]:

$${\text{KI}}_{\left(X\right)}=100\;N+100\;\frac{\mathrm{Log}\;t_{R\left(X\right)}-\mathrm{Log}\;t_{R\left(N\right)}}{\mathrm{Log}\;t_{R\left(N+1\right)}-\mathrm{Log}\;t_{R\left(N\right)}}$$

The cluster analysis was performed using the FactoMineR (Factor analysis and data Mining with R) package [43] in R (4.3.2) software for all the measured data regarding the content of each essential oil component obtained by SDSE-Hexane methods. A 2-dimensional plot was generated using the ggpubr R package. Two multivariate analyses, Principal Component Analysis (PCA) and Cluster Analysis, were performed to clarify the relationship among the component variables. PCA was employed to examine the differentiation of characteristic compounds found in the leaf essential oils of different *Cinnamomum* species. PCA is a type of multivariate technique that aims to reduce the dimensionality of data by transforming a set of related variables into a new set of independent variables, referred to as principal components (PCs). These PCs are created as linear combinations of the original variables and capture as much variation as possible [44]. By applying PCA to the content information of leaf essential oil components, a two-dimensional scattered plot was generated. The first principal component (PC1) represents the direction of maximum variability in the data, while the second principal component (PC2) captures the succeeding highest amount of variation. Each principal component is independent and orthogonal to the others. Through this analysis, the most significant characters contributing to the differentiation of *Cinnamomum* species were identified. The unweighted pair group method with arithmetic mean (UPGMA) analysis was also conducted to define the cluster. The UPGMA dendrogram was generated based on Euclidean distance as the measure criterion of similarity.

## 3 Results

### 3.1 Stream distillation with hexane extraction yields the highest chemical compounds for all three species

Most methods of extracting essential oil require larger sample pieces (over 200 g) and a lengthy extraction time to obtain sufficient quantity for gas chromatography-mass spectrometry (GC/MS) analysis [2,[45], [46], [47]]. We successfully established a method to extract essential oil using only 25 g of leaves, followed by evaluating the best extraction method. Our comparison included steam distillation (SD) alone and SD combined with solvent extraction (SDSE). Different solvents include acetone (SDSE-Acetone), ethyl acetate (SDSE-EA), and hexane (SDSE-Hexane). The average number of peaks from different extraction methods with 6-h distilled time in all three species ranged from 10 to 62.6. The lowest number was found in *C. camphora* (10.0 ± 0.71) using SD alone, while the highest number of peaks was found in *C. kanehirae* (62.6 ± 1.82) using SDSE-Hexane extraction (Appendix 1). Notably, the number of peaks identified from SDSE methods was 2.4–6.3 times higher than that of SD extraction.

**Fig. 2:**
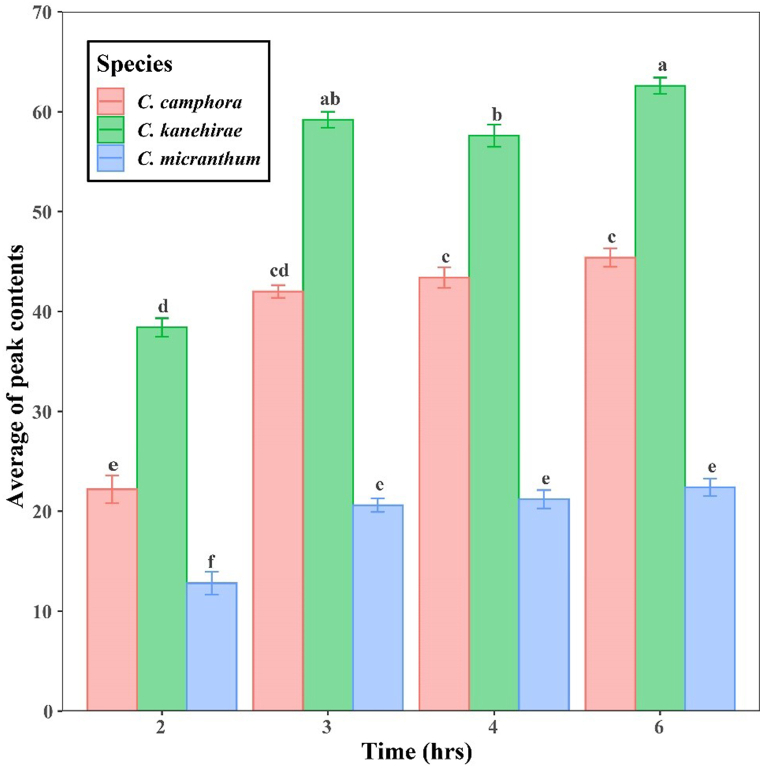
The average peak contents of *C. kanehirae*, *C. micranthum*, and *C. camphora* at different distillation times using SDSE-Hexane. Each column represents the mean of five values ± standard error. Statistical significance was determined by one-way ANOVA and Tukey simultaneous tests to compare the means. A significance level of *p* < 0.01 was observed. The presence of dissimilar letters following a bar denotes the existence of statistically significant differences within a single species across varied distillation times, ascertained by means of the Tukey test.

SDSE-Hexane yielded the highest number of identifiable peaks, so we further tested different extraction times using the SDSE-Hexane method. Overall, 2-h of distilled time resulted in significantly lower numbers of peaks in all three species compared to 3-h and above (Appendix 1). However, no significant differences existed between 3-, 4- and 6-h of distilled time (Fig. 2). These findings suggested that the maximum extraction efficiency of our experiments was achieved at 3 h of distillation time.

### 3.2 Chemotypes are significantly different among the three species

The chemical composition of the leaves of *C. kanehirae*, *C. micranthum*, and *C. camphora* was investigated by GC/MS analysis after being extracted by SDSE-Hexane for 3 h. The results revealed that the content of aromatic compounds, specifically terpenes including monoterpenes (MH), oxygenated monoterpenes (OM), sesquiterpenes (SH), and oxygenated sesquiterpenes (OS) varied among samples (Appendix 2 and 3). Overall, the average terpene contents in *C. camphora* and *C. kanehirae* were over 90 %, while those in *C. micranthum* were the lowest (58.6 %) among the three species. Based on the total ion chromatogram generated by GC/MS in all samples of three species, we identified three chemotypes in *C. camphora*, three chemotypes in *C. kanehirae*, and one chemotype in *C. miranthum* (Table 2, Appendix 4).

**Table 2:** The relative content of chemical compounds and chemotypes in leaf essential oils obtained by SDSE-Hexane methods of the three species from different geographic regions. SE: the southeastern region, NW: the northwestern region, WC: the west-central region, SW: the southwestern region.

Species	Chemotype	Chemical compounds	Mean (Minimum-Maximum)	Sample size	Region	Population
*C. kanehirae*	Linalool	Linalool	52.9 % (24.9–85.2 %)	29	SE	CK-TML-01-16、CK-TML-18-20、CK-TML-23、CK-LJ-01、CK-LJ-05-07、CK-LJ-12 -14
					NW	CK-DL-02、CK-DL-03
	Linalool/Eucalyptol	Linalool	39.1 % (24.3–47.1 %)	4	SE	CK-TML-21-22、CK-TML-24、CK-LJ-08
		Eucalyptol	16.6 % (11.2–20.7 %)			
	Linalool/Safrole	Linalool	39.3 % (32.7–45.7 %)	7	NW	CK-DL-01、CK-DL-06-10、CK-DL-12
		Safrole	9.2 % (5.2–23.7 %)			
*C. micranthum*	–	–	–	27	NW	CM–FS–01、CM–FS–02、CM–FS–03、CM–FS–04、CM–FS–05、CM–FS–06、CM–FS–08、CM–FS–09、CM–FS–10、CM–FS–11、CM–FS–12
					WC	CM-LHC-01、CM-LHC-03-04、CM-LHC-06-11、CM-LHC-14-20
*C. camphora*	Camphor	Camphor	66.8 % (48.0–75.3 %)	24	NW	CC–HC–01、CC-HC-05、CC-HC-08-09、CC-HC-11
					WC	CC-LHC-02
					SE	CC-YL-02-03、CC-YL-05、CC-YL-07-08、CC-YL-10、CCV-CG-01、CCV-CG-04-07、CCV-CG-09-13、CCV-CG-15、CCV-TML-02
	Linalool	Linalool	76.9 % (66.9–84.0 %)	8	NW	CC–HC–02、CC-HC-04、CC-HC-12
					SW	CCV-KT-03、CCV-KT-07-08、CCV-KT-10、CCV-LR-02
	Sesquiterpene	Nerolidol 2	25.5 % (16.5–47.4 %)	12	NW	CC–HC–03、CC-HC-06-07、CC-HC-10
					SE	CC-YL-01、CC-YL-04、CC-YL-06、CC-YL-09
					WC	CC-LHC-01、CC-LHC-03-05
		3-Methyl-2-butenoic acid, tridec-2-ynyl ester	36.5 % (0.1–58.3 %)			

In *C. kanehirae*, the highest content of terpenes was OM (37.2–89.1 %), followed by OS (7.2–45.0 %), SH (0.9–24.5 %), and MH (0.2–7.0 %) (Appendix 2 and 3). Total ion chromatogram (TIC) showed that the main components of essential oil obtained by SDSE-Hexane methods were eucalyptol (Kovats indices (KI) = 1033), linalool (KI = 1106) and safrole (KI = 1298) (Appendix 4). Based on this, three different chemotypes were identified: linalool type (with an average linalool content of 52.9 %), linalool/eucalyptol type (with an average content of linalool and eucalyptol for 39.1 and 16.6 %, respectively), and linalool/safrole type (with an average content of linalool and safrole for 39.3 and 9.2 %, respectively) (Table 2).

In *C. micranthum*, the highest content of terpenes was also OM (14.9–57.7 %), followed by the other compounds (29.3–74.0 %) and OS (7.9–23.0 %), but MH and SH were not detected in this species (Appendix 2 and 3). TIC showed that the main components were decanal (KI = 1218), nonanoic acid (KI = 1289), n-decanoic acid (KI = 1402), and undecanoic acid (KI = 1492) (Appendix 4). It is noteworthy that *C. micranthum* does not exhibit different chemotypes. Additionally, the TIC plot of *C. micranthum* is distinct from those of *C. kanehirae* and *C. camphora*.

In *C. camphora*, OM had the highest content (3.8–90.9 %), followed by OS (1.6–88.1 %), SH (1.9–28.3 %), other compounds (0–10.8 %), and MH (0–5.6 %) (Appendix 2 and 2), and TIC showed that the main components of essential oil obtained by SDSE-Hexane methods were linalool (KI = 1106), camphor (KI = 1187), safrole (KI = 1298), nerolidol 2 (KI = 1582), and 3-methyl-2-butenoic acid (KI = 1787) (Appendix 4). Based on the content of these components, *C. camphora* could be classified into three chemotypes: camphor type (average camphor content of 66.8 %), linalool type (average linalool content of 76.9 %), and sesquiterpene type (average Nerolidol 2/3-Methyl-2-butenoic acid content of 25.5/36.5 %) (Table 2).

Most of the *C. kanehirae* samples were identified as linalool chemotypes, including 27 samples from southeast Taiwan and 2 samples from northwest Taiwan (Table 2). In addition, 4 and 7 samples were linalool/eucalyptol and linalool/safrole chemotypes, and were from southeast and from northwest Taiwan, respectively. Conversely, samples of *C. camphora* do not show geographic distinctiveness in all three chemotypes.

### 3.3 Multivariate analysis of leaf essential oil composition

**Fig. 3:**
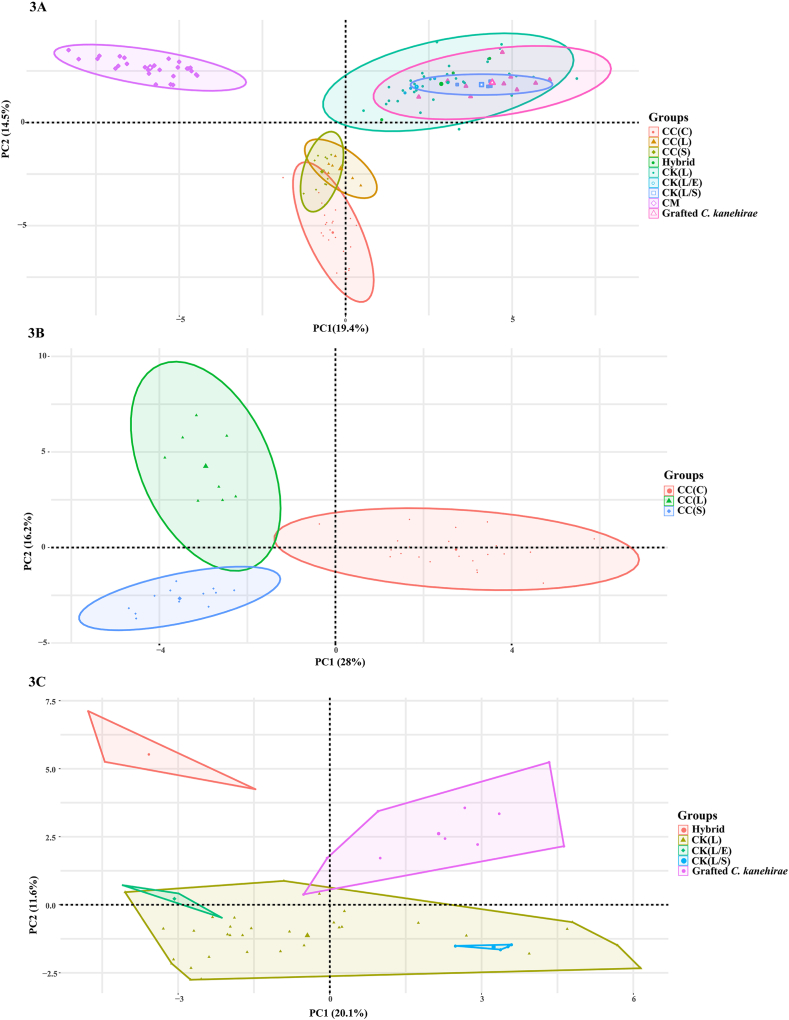
The results of principal component analysis (PCA) are based on the content information of leaf essential oil components obtained by SDSE-Hexane methods. (3A) The PCA of *C. kanehirae*, *C. micranthum* and *C. camphora*, grafted *C. kanehirae*, and hybrid. (3B) The PCA of different chemotypes of *C. camphora*. (3C) The PCA of different chemotypes of *C. kanehirae*. CC(C): *C. camphora* (camphor type); CC(L): *C. camphora* (linalool type); CC(S): *C. camphora* (sesquiterpene type); CK(L): *C. kanehirae* (linalool type); CK(L/E): *C. kanehirae* (linalool/eucalyptol type); CK(L/S): *C. kanehirae* (linalool/safrole type); CM: *C. micranthum*.

We further asked the question of whether the components of essential oil from three different species (*C. kanehirae*, *C. micranthum*, and *C. camphora*) obtained by SDSE-Hexane methods are grouped based on their respective species. PCA analysis was conducted using the GC/MS data of the essential oil components from the three species. The analysis revealed that the first three eigenvalues were 13.6, 10.2, and 5.1, with cumulative variance percentages of 19.4 % (PC1), 14.6 % (PC2), and 7.3 % (PC3), respectively (Appendix 5). This accounted for a cumulative variance of 41.22 %. The contributions of the selected variables to PC1 and PC2 were displayed in Appendix 6 and Appendix 7. These contributions are expressed as percentages, where a higher contribution of a variable to the principal component indicates a greater impact of the variable on developing that component. A two-dimensional coordinate plot was generated using the first two principal components. The PCA results revealed that the three species displayed distinct clustering, indicating that their unique chemotype compositions corresponded with the conventional morphological classification results (Fig. 3A).

The PCA result also showed that grafted *C. kanehirae* were grouped together with natural *C. kanehirae* populations (Fig. 3A). For *C. camphor*a, samples were clustered based on the chemotypes (Fig. 3B). However, samples of *C. kanehirae* could not be grouped based on chemotypes This is probably because all chemotypes have linalool as their major compound (averaging 52.9 %, 39.1 %, and 39.3 % for three chemotypes, respectively) (Fig. 3C).

**Fig. 4:**
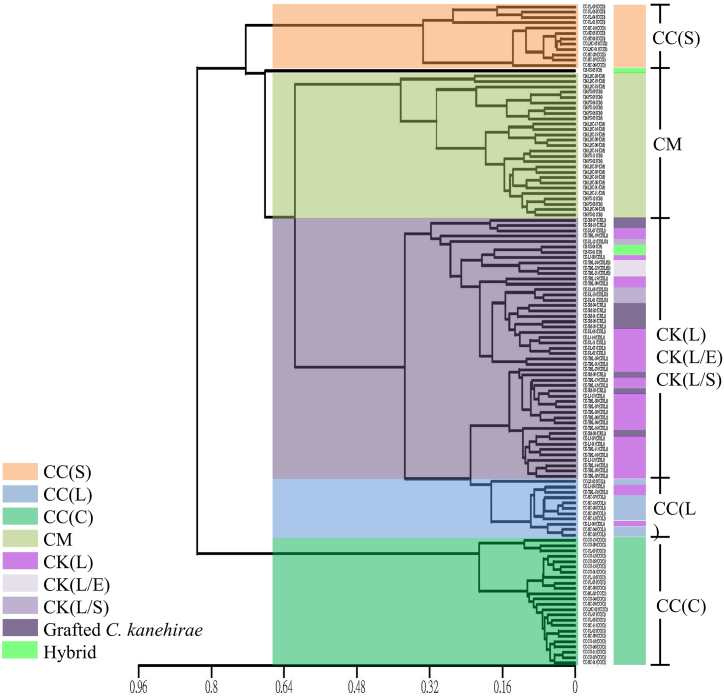
The UPGMA dendrogram was constructed by Cluster Analysis based on the main constituents of leaf essential oils obtained by SDSE-Hexane methods. CC(C): *C. camphora* (camphor type); CC(L): *C. camphora* (linalool type); CC(S): *C. camphora* (sesquiterpene type); CK(L): *C. kanehirae* (linalool type); CK(L/E): *C. kanehirae* (linalool/eucalyptol type); CK(L/S): *C. kanehirae* (linalool/safrole type); CM: *C. micranthum*; CH: hybrid.

Furthermore, clustering analysis using the UPGMA dendrogram was performed, which divided the samples into four distinct groups: *C. camphora* sesquiterpene type (CC(S)), *C. micranthum* (CM), *C. kanehirae* and *C. camphora* linalool type (CK and CC(L)) and *C. camphora* camphor type (CC(C)) (Fig. 4). We found that the linalool type of *C. kanehirae* and *C. camphora* were grouped, most likely due to the large proportion of linalool in their essential oil components obtained by SDSE-Hexane methods. In addition, hybrid samples between *C. kanehirae* and *C. camphora* were grouped with *C. kanehirae*. A similar situation was observed in the grafted *C. kanehirae*. The results were in line with PCA analyses.

## 4 Discussions

This study conducted GC/MS analysis and evaluated the extraction efficiency using the number of the identified peak, comparing the SD and SDSE extraction methods to optimize the extraction efficiency for small sample volumes. Most *Cinnamomum* essential oil studies used the SD method for 6–8 h, followed by GC/MS analysis of the chemical composition diversity [2,[45], [46], [47]]. In this study, SD, SDSE-Acetone, SDSE-EA, and SDSE-Hexane methods were used to extract the essential oils of smaller amount (25 g) of *C. kanehirae*, *C. micranthum*, and *C. camphora* leaves. The results revealed that the number of peaks from SDSE extraction methods was 2.4–6.3 times higher than those from SD. This suggests that the chemical components of the leaf's essential oil are transferred from the aqueous layer (high polarity) to the organic layer (low polarity) during liquid-liquid extraction, which resulted in the increase of essential oil purity and a more diverse range of detected chemical components.

For the chemical composition of *C. kanehirae*, *C. micranthum*, and *C. camphora* leaves, SDSE-Hexane was used for extraction as it resulted in the highest number of essential oil peaks. Using the SDSE-Hexane method, we extracted for 2, 3, 4, and 6 h. The results showed that the number of identifiable peaks increased with distillation time, but no statistically significant increase occurred after 3 h. Hence, we recommended 3 h with the SDSE-Hexane method to reach the maximum distillation efficiency.

In *C. kanehirae*, the most abundant chemical composition was found to be OM, consistent with the results of the previous study by Cheng et al. (2015) [2]. The TIC profile of *C. micranthum* is utterly different from that of *C. kanehirae* and *C. camphora*. These findings align with previous studies of distinct chemical characteristics between *C. kanehirae* and *C. micranthum* [14,[28], [29], [30]]. Although Yang et al. (2022) [31] used DNA molecular markers to reconstruct the phylogeny of the *Cinnamomum* genus and merged *C. kanehirae* with *C. micranthum* as a single species, this study shows apparent differences in leaf essential oil chemical composition between the two species obtained by SDSE-Hexane methods. Therefore, integrating morphological, chemical, and molecular biological information, the systematic classification of the *Cinnamomum* genus still requires further discussion.

In this study, we identified three chemotypes of *C. kanehirae*. In the linalool/safrole type, we observed a high concentration of safrole in the leaves (average of 9.2 %, range of 5.2–23.7 %). Safrole was typically found in *C. kanehirae* branches but was not reported in the leaves previously [2]. We detected safrole in 7 out of 40 samples (content exceeding 5 %). Among the 7 samples, 6 of them were collected in Hsinchu (samples CK-DL-01, 06, 07, 08, 10, and 12 with composition ranging from 6.91 to 30.3 %) and 1 collected in Taitung (CK-LJ-12 with composition of 7.9 %). Notably, most samples with high Safrole content were found in the NW region, indicating that it might be a regional chemotype. This also provides a reference for future regulations for the species.

Different chemical types of *C. camphora* were found specifically in different regions of Taiwan. *C. camphora* is mainly distributed in the western and central mountainous areas of Taiwan and is referred to as the western camphor trees. In addition, those distributed in the southern and eastern coastal regions of Taiwan are known as the eastern camphor trees. Eastern camphor trees were previously referred to as *C. camphora* var. *nominalis*. However, the *C. camphora* var. *nominalis* closely resembles *C. camphora* and can only be distinguished by its geographic location. Therefore, many studies classify them as the same species [26,27]. This study found that samples from the NW region contained three chemical types (camphor, linalool and sesquiterpene type). However, two chemotypes were identified in the samples from WC and SE regions (camphor and sesquiterpene types), and the SW population is restricted to the linalool chemotype. As the number of samples from the SW region was limited, a further study to confirm this result is required. While chemotypes were unable to differentiate between eastern and western camphor trees, samples from the NW and SW regions exhibited a unique Linalool chemotype that could be distinguished from those of the WC and SE regions. Furthermore, the NW and SW regions could be further differentiated based on the presence of camphor and sesquiterpene chemotypes. We concluded that essential oil chemical composition analysis not only assists in defining species boundaries among closely related *Cinnamomum* species but also provides a reference for future germplasm of related *Cinnamomum* species.

The terpenoid compounds OM and OS are indicative of *C. kanehirae* and *C. camphora*, respectively. These compounds are derived from two biosynthetic pathways, the MVA and MEP pathways (reviewed and summarized in Appendix 8) [2,[48], [49], [50]]. Camphor, found in the camphor type of *C. camphora*, is derived from GPP (geranyl diphosphate) through a cyclization reaction to form (+)-bornyl-d-phosphate and then converted to camphor. Nerolidol, found in the sesquiterpene type of *C. camphora*, is derived from IPP (isopentenyl diphosphate) and is converted to FPP (farnesyl diphosphate) by FPP synthase. Linalool, found in the linalool type of *C. camphora* and *C. kanehirae*, is derived from GPP and catalyzed by terpene synthases (geranyl diphosphate synthase, GPPS, and linalool synthase, LINS) to convert to linalool. Since the indicator component (linalool) is derived from the same biosynthetic pathway, the composition of *C. kanehirae* is more similar to the linalool type of *C. camphora*. On the other hand, based on the theory that linalool can be converted to terpineol, Fujita proposed that the Taiwanese *Cinnamomum* species originated from *C. camphora* var. *linaloolifera* (containing l-linalool) and then evolved and diverged to *C. kanehirae* (containing d-terpineol-(4)) and *C. micranthum* (containing decyl aldehyde and penradecyl aldehyde) [[36], [37], [38]]. Based on the clustering analysis in this study and the theory that linalool can be converted to terpineol, both support the idea that *C. kanehirae* is more closely related to *C. camphora*.

This study investigated whether the chemical composition of grafted *C. kanehirae* differs from that of natural *C. kanehirae*. The PCA and UPGMA results showed that grafted and natural *C. kanehirae* and *C. camphora* of the linalool type belonged to the same cluster. Furthermore, the chemical composition analysis did not detect any characteristic components of *C. camphora* (camphor and nerolidol) in grafted *C. kanehirae*. The essential oil chemical classification method indicated that the mature leaves in grafted *C. kanehirae* are not affected by rootstock, which is consistent with the morphological classification.

Regarding the chemical composition identification of natural hybrid *C. kanehirae*, the CA and PCA analyses of the essential oil from the natural hybrid species (*C. hybrid*) showed that they belong to the same cluster as the *C. kanehirae* population. Nevertheless, a trace amount of camphor was detected, indicating that the parental species might influence the chemical composition of hybrids. Wu et al. (2020) identified three types of natural hybrids, F1, F2 (F1 x F1), and backcross (B x 0: F1 x *C. kanehirae*; B x 1: F1 x *C. camphora*), with *C. kanehirae* being the most common maternal parent. Natural hybrids exhibited significant genetic differentiation from both *C. kanehirae* and *C. camphora*, and their essential oil composition may be between the two parental species [28]. The collected natural hybrids in this study exhibited a chemical composition biased towards *C. kanehirae*, indicating it was likely the maternal parent. The amount of camphor detected in the natural hybrids may be related to the hybrid or backcross times with the *C. camphora*.

Taken together, this study provides an essential reference for future research on genetic variation in *Cinnamomum*. It also offers valuable insights into species identification, diversity, and breeding of *Cinnamomum*. Moreover, the findings have significant implications and value in related fields.

## Funding

This research was funded by the Pingtung County Central Laboratory to W·H·C., by the 10.13039/100013035Taiwan Forestry Research Institute to L.P.J, and by the 10.13039/100020595National Science and Technology Council, Taiwan [10.13039/501100004663MOST 108-2621-B-110-003-MY3, 10.13039/501100004663MOST 109-2313-B-110-005, 10.13039/501100004663MOST 111-2621-B-110-001 and 10.13039/100020595NSTC 112-2621-B-110-001-MY3] to Y·C.C. and by partial financing (the Higher Education Sprout Project) of 10.13039/100007844NSYSU.

## Data availability statement

Data associated with this study has not been publicly available in any repository. The article includes the data in the Article/Supplementary Material/Referenced. Further inquiries can be directed to the corresponding author.

## CRediT authorship contribution statement

**Wen-Hui Chen:** Writing – review & editing, Writing – original draft, Visualization, Validation, Software, Resources, Methodology, Investigation, Funding acquisition, Formal analysis, Data curation, Conceptualization. **Ya-Zhu Ko:** Writing – review & editing, Writing – original draft, Visualization, Validation, Software, Methodology, Investigation, Formal analysis, Data curation. **Hsiu-Chun Chang:** Data curation. **Chui-Shiang Chang:** Data curation. **Kuo-Hsiang Hung:** Writing – original draft, Data curation. **Huie-Chuan Shih:** Writing – original draft, Data curation. **Li-Ping Ju:** Writing – review & editing, Writing – original draft, Resources, Data curation, Conceptualization. **Meng-Shin Shiao:** Writing – review & editing, Writing – original draft, Validation, Data curation, Conceptualization. **Yu-Chung Chiang:** Writing – review & editing, Writing – original draft, Visualization, Validation, Supervision, Resources, Project administration, Methodology, Funding acquisition, Data curation, Conceptualization.

## Declaration of competing interest

The authors declare the following financial interests/personal relationships which may be considered as potential competing interests: Yu-Chugn Chiang is the Section Editor of Plant Biology.

## Footnotes

Appendix A Supplementary data to this article can be found online at https://doi.org/10.1016/j.heliyon.2024.e30628.
