# Supplementary material for: Comparative chemical profiling of leaf essential oils from Cinnamomum kanehirae and related species using steam distillation and solvent extraction: Implications for plant-based classification
Source: Heliyon. 2024 May 3;10(9):e30628. doi: 10.1016/j.heliyon.2024.e30628 (PMC11079393; doi:10.1016/j.heliyon.2024.e30628)
Supplement: Multimedia component 1 [file mmc1.docx]

**Supplementary data**

Appendix 1. The average number of identifiable peaks for solvent extracted from the leaves of *C. kanehirae*, *C. micranthum*, and *C. camphora* using different methods and distillation times. The value is the mean (M) ± standard deviation (SD) based on quintuplicate measurements.

| **Extraction** | **Distillation Time (hrs)** | ***C. kanehirae*** | ***C. micranthum*** | ***C. camphora*** |
| --- | --- | --- | --- | --- |
| SD | 6 | 10.0±0.71 | 9.2±0.84 | 10.0±0.71 |
|  |  |  |  |  |
| SDSE-Acetone | 6 | 27.4±3.44 | 9.4±0.55 | 22.2±0.84 |
|  |  |  |  |  |
| SDSE-EA | 6 | 52.6±3.71 | 9.4±0.55 | 24.2±2.49 |
|  |  |  |  |  |
| SDSE-Hexane | 6 | 62.6±1.82 | 22.4±1.95 | 45.4±2.07 |
|  | 2 | 38.4±2.07 | 12.8±2.59 | 22.2±3.11 |
|  | 3 | 59.2±1.79 | 20.6±1.52 | 42.0±1.41 |
|  | 4 | 57.6±2.51 | 21.2±2.05 | 43.4±2.30 |

Appendix 2. Constituents and relative contents (%) of 111 leaf essential oils from *C. kanehirae*, *C. micranthum*, and *C. camphora* obtained by SDSE-Hexane methods. "-" indicates that the compound was undetected, and 0.00 indicates that the relative content was less than 0.00%.

| **No.** | **Compounds** | | **KI^a^** | **RT**  **(min)** | ***C. kanehirae*** | | | ***C. micranthu*** | ***C. camphor*** | | |
| --- | --- | --- | --- | --- | --- | --- | --- | --- | --- | --- | --- |
|  |  |  |  |  | **Linalool type** | **linalool/eucalyptol type** | **linalool /safrole type** | **one type** | **camphor type** | **Linalool type** | **sesquiterpene type** |
|  |  |  |  |  | **N=29** | **N=4** | **N=7** | **N=27** | **N=24** | **N=8** | **N=12** |
| 1 | β-Terpinene | | 934 | 7.73 | - | - | - | - | 0.8 | 0.1 | - |
| 2 | β-Myrcene | | 994 | 9.92 | - | - | - | - | 0.6 | 0.1 | - |
| 3 | Octanal | | 1008 | 10.51 | - | - | - | 0.7 | 0.5 | 0.1 | - |
| 4 | α-Phellandrene | | 1011 | 10.63 | 0.2 | 0.3 | 1.5 | - | - | - | - |
| 5 | 1,3,8-p-Menthatriene | | 1029 | 11.43 | 1.3 | 0.4 | 1.9 | - | - | 0.7 | - |
| 6 | Eucalyptol | | 1033 | 11.61 | 1.00 | 16.6 | 1.3 | 0.8 | 3.9 | 0.1 | 0.8 |
| 7 | Isoterpinolene | | 1088 | 14.06 | 0.3 | 0.4 | 0.2 | - | 0.5 | - | - |
| 8 | Linalool | | 1106 | 14.83 | 52.9 | 39.1 | 39.3 | 2.9 | 1.5 | 77.0 | 2.1 |
| 9 | Nonanal | | 1112 | 15.10 | - | - | - | 3.9 | - | - | - |
| 10 | (+)-2-Bornanone | | 1150 | 16.85 | - | - | - | 0.7 | - | - | 2.6 |
| 11 | 3-Cyclohexen-1-ol | | 1184 | 18.42 | 3.8 | 4.1 | 2.0 | 3.6 | - | - | - |
| 12 | Camphor | | 1187 | 18.53 | - | - | - | - | 66.8 | 2.9 | - |
| 13 | Endo-Borneol | | 1194 | 18.86 | 0.1 | 0.1 | 0.2 | - | 1.5 | 0.4 | - |
| 14 | Terpinen-4-ol | | 1200 | 19.15 | 3.6 | 3.0 | 2.0 | 0.6 | 2.4 | 0.2 | 0.6 |
| 15 | α-Terpineol | | 1202 | 19.23 | 1.2 | 6.9 | 0.4 | - | - | - | - |
| 16 | 4(10)-Thujen-3-ol, stereoisomer | | 1209 | 19.57 | 0.1 | 0.1 | 0.6 | - | 1.4 | 0.7 | - |
| 17 | Decanal | | 1218 | 19.97 | - | - | - | 20.0 | - | 0.1 | - |
| 18 | (E,E)-2,6-Dimethyl-3,5,7-octatrien-2-ol | | 1223 | 20.18 | - | - | - | - | - | 1.1 | - |
| 19 | Isoartemisia ketone | | 1232 | 20.59 | 1.0 | 2.1 | 0.2 | - | 0.2 | 1.2 | 0.8 |
| 20 | 9-Dodecyn-1-ol | | 1239 | 20.92 | - | - | - | - | - | 0.3 | - |
| 21 | Citral | | 1248 | 21.33 | 0.3 | 0.5 | 0.1 | - | 0.1 | 0.1 | - |
| 22 | Linalyl acetate | | 1259 | 21.79 | 0.2 | 0.2 | 0.8 | - | - | - | - |
| 23 | cis-Geraniol | | 1260 | 21.86 | - | - | - | - | 0.2 | 0.4 | - |
| 24 | Nonanoic acid | | 1289 | 23.17 | - | - | - | 9.6 | - | - | - |
| 25 | Borneol | | 1292 | 23.28 | - | - | - | - | 1.7 | 0.4 | - |
| 26 | Safrole | | 1298 | 23.57 | 0.1 | - | 9.2 | - | - | - | 2.9 |
| 27 | Elemene isomer | | 1338 | 25.28 | - | - | - | - | 0.4 | 0.2 | 0.4 |
| 28 | cis-4-Propenylguaiacol | | 1358 | 26.15 | - | - | - | - | 0.2 | 0.1 | 0.4 |
| 29 | Copaene | | 1379 | 27.08 | 0.6 | 0.2 | 0.5 | - | 0.1 | 0.1 | 0.5 |
| 30 | Germacrene B | | 1394 | 27.72 | 0.3 | 0.1 | 0.4 | - | 0.4 | 0.3 | 0.4 |
| 31 | n-Decanoic acid | | 1402 | 28.06 | - | - | - | 13.4 | - | - | - |
| 32 | Methyleugenol | | 1407 | 28.27 | - | - | - | - | 0.3 | 0.1 | - |
| 33 | Caryophyllene | | 1424 | 28.96 | 2.3 | 0.5 | 2.0 | - | 4.1 | 2.1 | 3.3 |
| 34 | trans-7-epi-Sesquisabinene hydrate | | 1440 | 29.63 | - | - | - | - | - | 0.2 | - |
| 35 | Spirojatamol | | 1453 | 30.19 | - | - | - | 2.3 | 1.7 | 0.5 | 1.5 |
| 36 | Humulene | | 1459 | 30.42 | 0.5 | 0.2 | 0.4 | - | 0.5 | 0.3 | - |
| 37 | γ-Muurolene | | 1480 | 31.30 | 0.3 | 0.2 | 0.5 | - | - | - | - |
| 38 | Germacrene D | | 1486 | 31.53 | 0.6 | 0.3 | 0.8 | - | 1.5 | 0.6 | 0.6 |
| 39 | δ-Guaijene | | 1492 | 31.77 | - | - | - | - | 1.7 | 1.0 | 1.0 |
| 40 | Undecanoic acid | | 1492 | 31.81 | - | - | - | 12.8 | - | - | - |
| 41 | cis-muurola-4(14),5-diene | | 1493 | 31.85 | 1.8 | 0.8 | 1.2 | - | - | - | - |
| 42 | 10-epi-γ-Eudesmol | | 1498 | 32.05 | 1.1 | 0.7 | 2.3 | - | 1.3 | 0.6 | 1.1 |
| 44 | Naphthalene | | 1523 | 33.00 | 2.0 | 1.7 | 3.6 | - | 0.2 | 0.2 | 0.5 |
| 45 | Ledene oxide-(II) | | 1548 | 34.00 | - | - | - | - | - | - | - |
| 46 | allohedycaryol | | 1553 | 34.20 | 0.1 | 0.1 | 1.0 | - | 0.2 | 0.3 | - |
| 47 | 5-Hydroxymethyl-1,3,3-trimethyl-2-(3-methyl-buta-1,3-dienyl)-cyclopentanol | | 1562 | 34.55 | - | - | - | 1.4 | - | - | - |
| 48 | 1,6,10-Dodecatrien-3-ol | | 1569 | 34.82 | - | - | - | 1.0 | 0.4 | 1.0 | - |
| 49 | 4aH-cycloprop[e]azulen-4a-ol | | 1573 | 34.95 | 1.7 | 1.1 | 0.6 | - | - | - | - |
| 50 | Nerolidol 2 | | 1582 | 35.33 | - | - | - | - | 1.1 | 2.0 | 24.6 |
| 51 | Germacrene D-4-ol | | 1586 | 35.46 | 5.1 | 4.2 | 6.1 | 6.4 | - | - | - |
| 52 | Guaiol acetate | | 1598 | 35.95 | - | - | - | 1.6 | - | - | - |
| 53 | 1H-Cycloprop[e]azulen-4-ol | | 1609 | 36.36 | 1.3 | 0.7 | 0.7 | - | - | - | - |
| 54 | Alloaromadendrene oxide-(1) | | 1612 | 36.47 | - | - | - | - | - | - | - |
| 55 | 2-Naphthalenemethanol | | 1626 | 36.99 | 3.6 | 2.6 | 1.3 | 2.4 | - | - | - |
| 56 | Epicubenol | | 1633 | 37.26 | 0.6 | 0.5 | 0.3 | - | - | - | - |
| 57 | τ-Cadinol | | 1653 | 37.99 | 1.5 | 1.0 | 4.3 | - | - | - | - |
| 58 | α-Cadinol | | 1666 | 38.47 | 4.8 | 5.7 | 4.3 | - | - | - | - |
| 59 | Isoaromadendrene epoxide | | 1678 | 38.91 | 0.7 | 0.8 | - | - | - | - | - |
| 60 | 5-Azulenemethanol | | 1679 | 38.94 | 1.1 | 0.5 | 1.8 | - | - | - | - |
| 61 | 1-Hexyl-2-nitrocyclohexane | | 1697 | 39.61 | - | - | - | 3.1 | - | - | - |
| 62 | Nerolidol | | 1706 | 39.94 | - | - | - | - | - | 2.3 | 8.0 |
| 63 | (R,S)-5-Ethyl-6-methyl-3E-hepten-2-one | | 1712 | 40.16 | - | - | - | 1.9 | - | - | - |
| 64 | ethanone | | 1739 | 41.11 | 0.2 | 0.1 | 1.1 | - | - | - | - |
| 65 | Formic acid | | 1754 | 41.64 | - | - | - | 1.5 | - | - | - |
| 66 | 3-Methyl-2-butenoic acid, tridec-2-ynyl ester | | 1787 | 42.81 | - | - | - | - | - | - | 45.0 |
| 67 | 2-Pentadecanone | | 1852 | 45.03 | - | - | - | - | - | - | - |
| 68 | 2-Acetoxy-1,1,10-trimethyl-6,9-epidioxydecalin | | 1853 | 45.07 | 0.3 | 0.2 | 0.1 | - | - | - | - |
| 69 | Phthalic acid, nonyl tridec-2-yn-1-yl ester | | 1866 | 45.52 | 0.2 | 0.4 | - | - | - | - | - |
| 70 | trans-Bisabolene epoxide | | 1874 | 45.77 | - | - | - | - | - | - | - |
| 71 | 1H-Benzocyclohepten-7-ol | | 1950 | 48.26 | - | - | - | 0.9 | - | - | - |
| 72 | Dibutyl phthalate | | 1961 | 48.62 | - | - | - | 0.5 | - | 0.1 | - |
| 73 | n-Hexadecanoic acid | | 1975 | 49.08 | 1.0 | 0.6 | 0.5 | 3.9 | 0.2 | 0.1 | - |
| 74 | Pregna | | 2090 | 52.70 | - | - | - | 0.4 | - | - | - |
| 75 | 2-Piperidinone | | 2192 | 55.25 | - | - | - | 3.6 | - | - | - |
| Monoterpenes  hydrocarbons (%) | | |  | Mean | 1.9 | 0.8 | 3.6 | 0.0 | 1.9 | 0.8 | 0.0 |
|  |  |  |  | Min-Max | 0.2-7.0 | | | 0.0 | 0.0-5.6 | | |
| Oxygenated  monoterpenes (%) | | |  | Mean | 64.0 | 72.4 | 55.3 | 44.0 | 78.1 | 84.01 | 16.7 |
|  |  |  |  | Min-Max | 37.2-89.1 | | | 14.9-57.7 | 3.8-90.9 | | |
| Sesquiterpenes  hydrocarbons (%) | | |  | Mean | 8.3 | 3.8 | 9.3 | 0.0 | 8.9 | 4.7 | 10.4 |
|  |  |  |  | Min-Max | 0.9-24.5 | | | 0.0 | 1.9-28.3 | | |
| Oxygenated  sesquiterpenes (%) | | |  | Mean | 22.0 | 18.2 | 23.7 | 14.6 | 4.7 | 6.8 | 70.8 |
|  |  |  |  | Min-Max | 7.2-45.0 | | | 7.9-23.0 | 1.6-88.1 | | |
| other (%) | | |  | Mean | 1.4 | 1.2 | 1.4 | 41.4 | 2.6 | 0.9 | 1.3 |
|  |  |  |  | Min-Max | 0.3-3.5 | | | 29.3-74.0 | 0.1-10.8 | | |
|  | | Total (%) | | | 97.7 | 96.5 | 93.2 | 100.0 | 96.3 | 97.3 | 99.2 |

^a^ Kovats index relative to n-alkanes (C8-C22) on a DB-5MS column.

N: Sample size; Min: Minimum; Max: Maximum


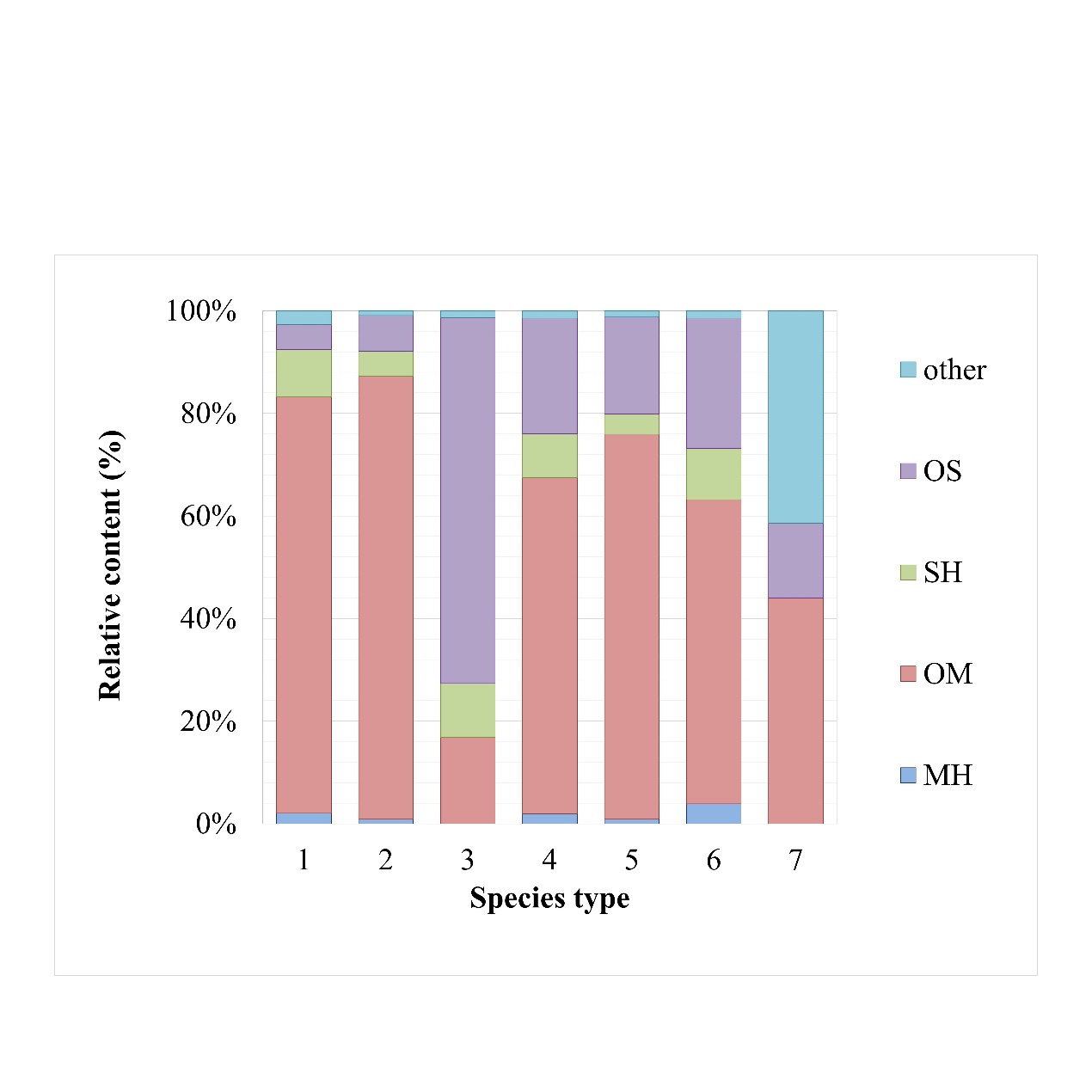


Appendix 3. Relative contents of volatile constituents in the essential oils from the leaves of *C. kanehirae*, *C. micranthum* and *C. camphora* obtained by SDSE-Hexane methods. 1: *C. camphora* - camphora type; 2: *C. camphora* - linalool type; 3: *C. camphora* - sesquiterpene type; 4: *C. kanehirae* - linalool type; 5: *C. kanehirae* - linalool/eucalyptol type; 6: *C. kanehirae* - linalool/ safrole type; 7: *C. micranthum*. MH: monoterpenes hydrocarbons; OM: oxygenated monoterpenes; SH: sesquiterpenes hydrocarbons; OS: oxygenated sesquiterpenes.


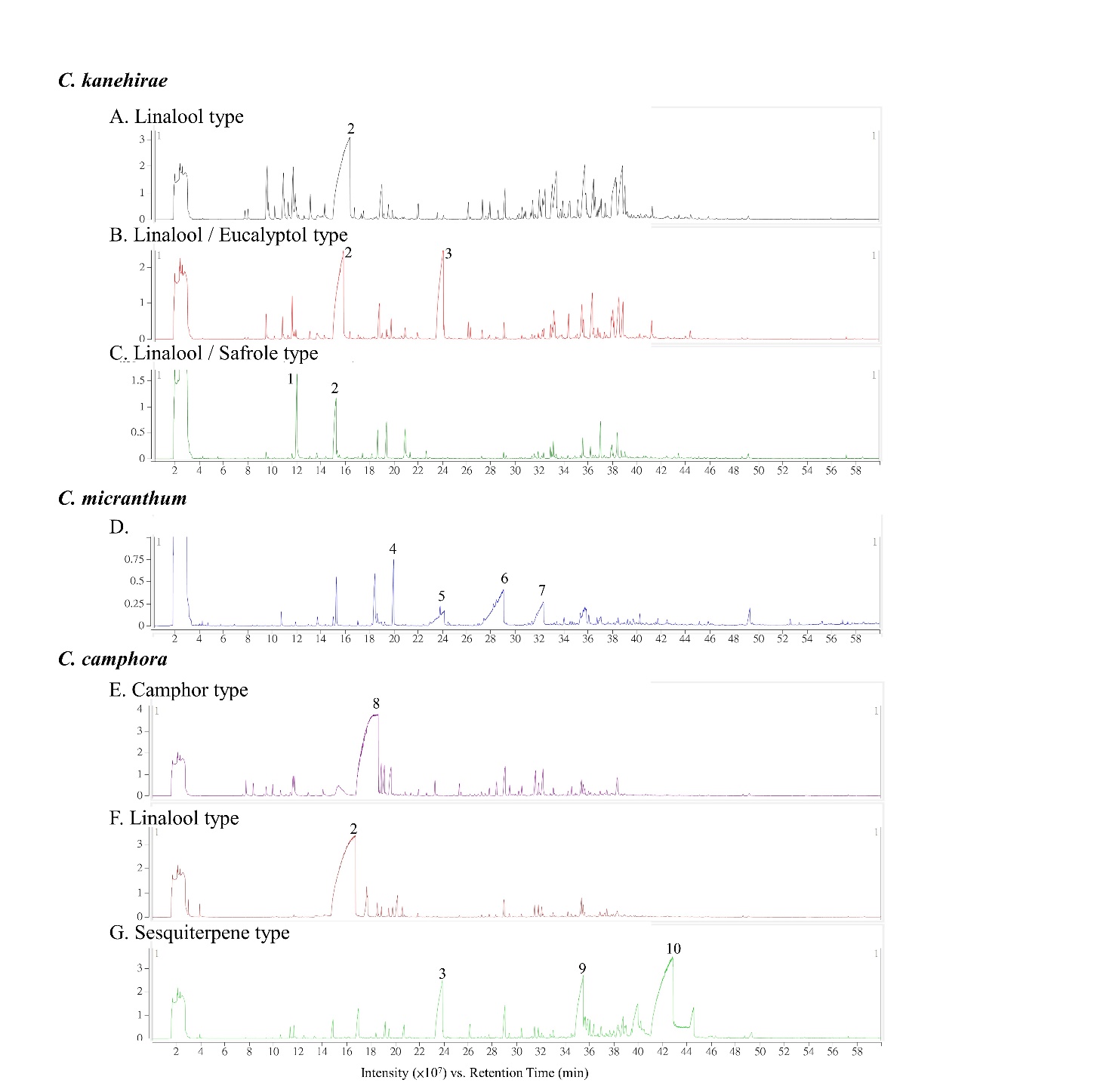


Appendix 4. The total ion chromatogram (TIC) of leaf essential oils from *C. kanehirae*, *C. micranthum* and *C. camphora* obtained by SDSE-Hexane methods. (A) *C. kanehirae*: linalool type; (B) *C. kanehirae*: linalool/ safrole type; (C) *C. kanehirae*: linalool/eucalyptol type; (D) *C. micranthum*; (E) *C. camphora*: camphora type; (F) *C. camphora*: linalool type; (G) *C. camphora*: sesquiterpene type. Chemical composition code: 1: eucalyptol; 2: linalool; 3: safrole; 4: decanal; 5: nonanoic acid; 6: n-decanoic acid; 7: undecanoic acid; 8: camphor; 9: nerolidol; 10: 3-methyl-2-butenoic acid


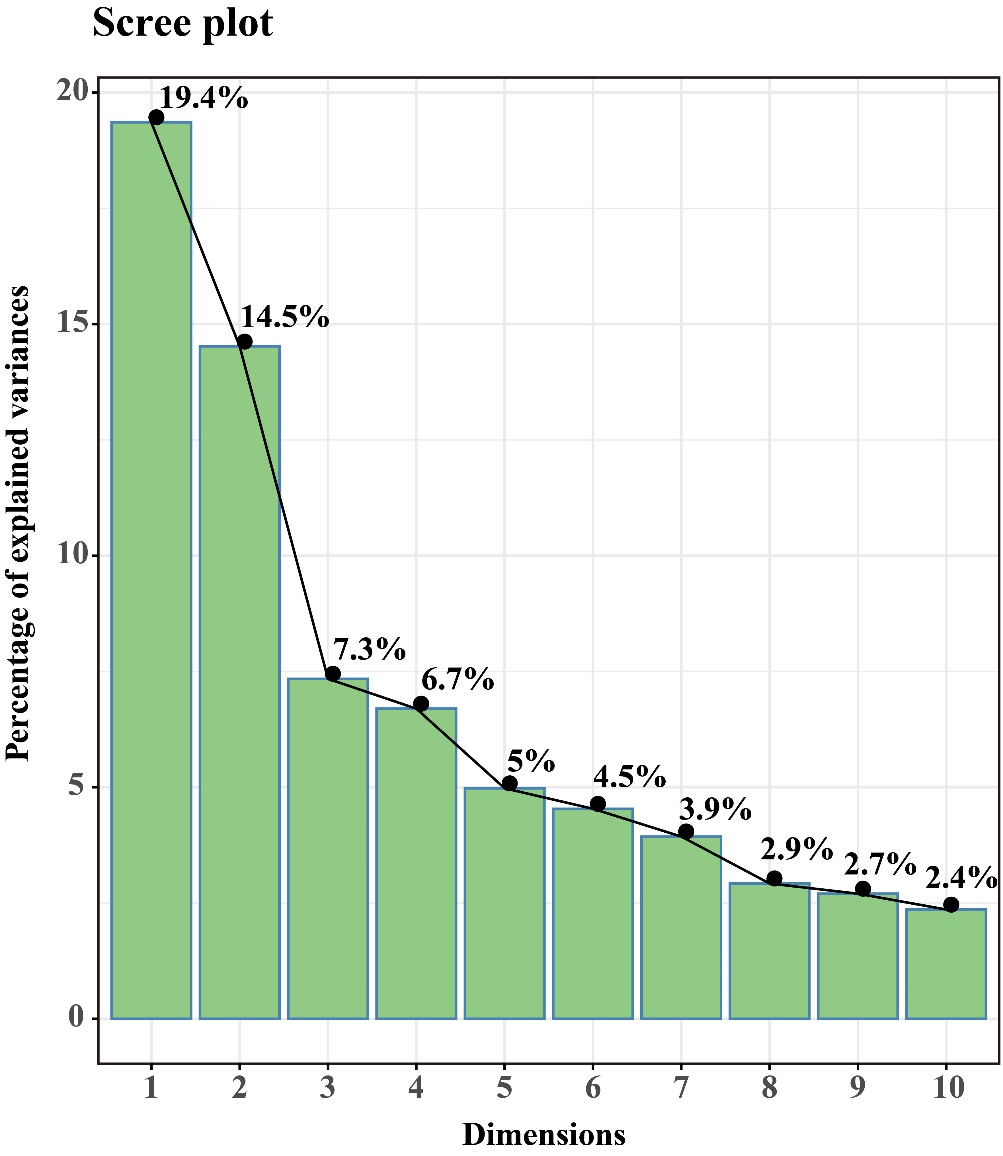
Appendix 5. The principal component analysis displays the eigenvalues of various principal components.


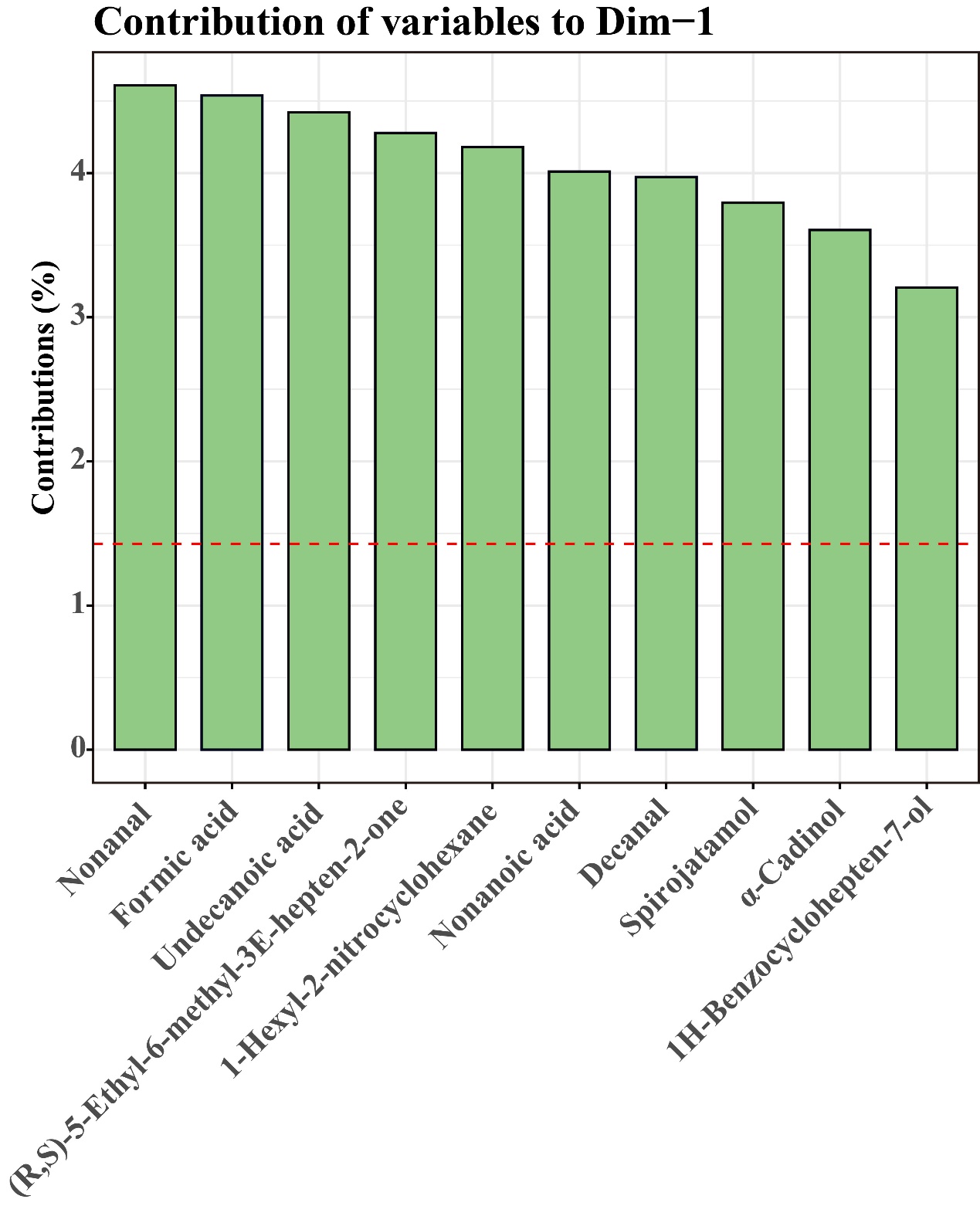
Appendix 6. The variables selected and their contribution to PC1.


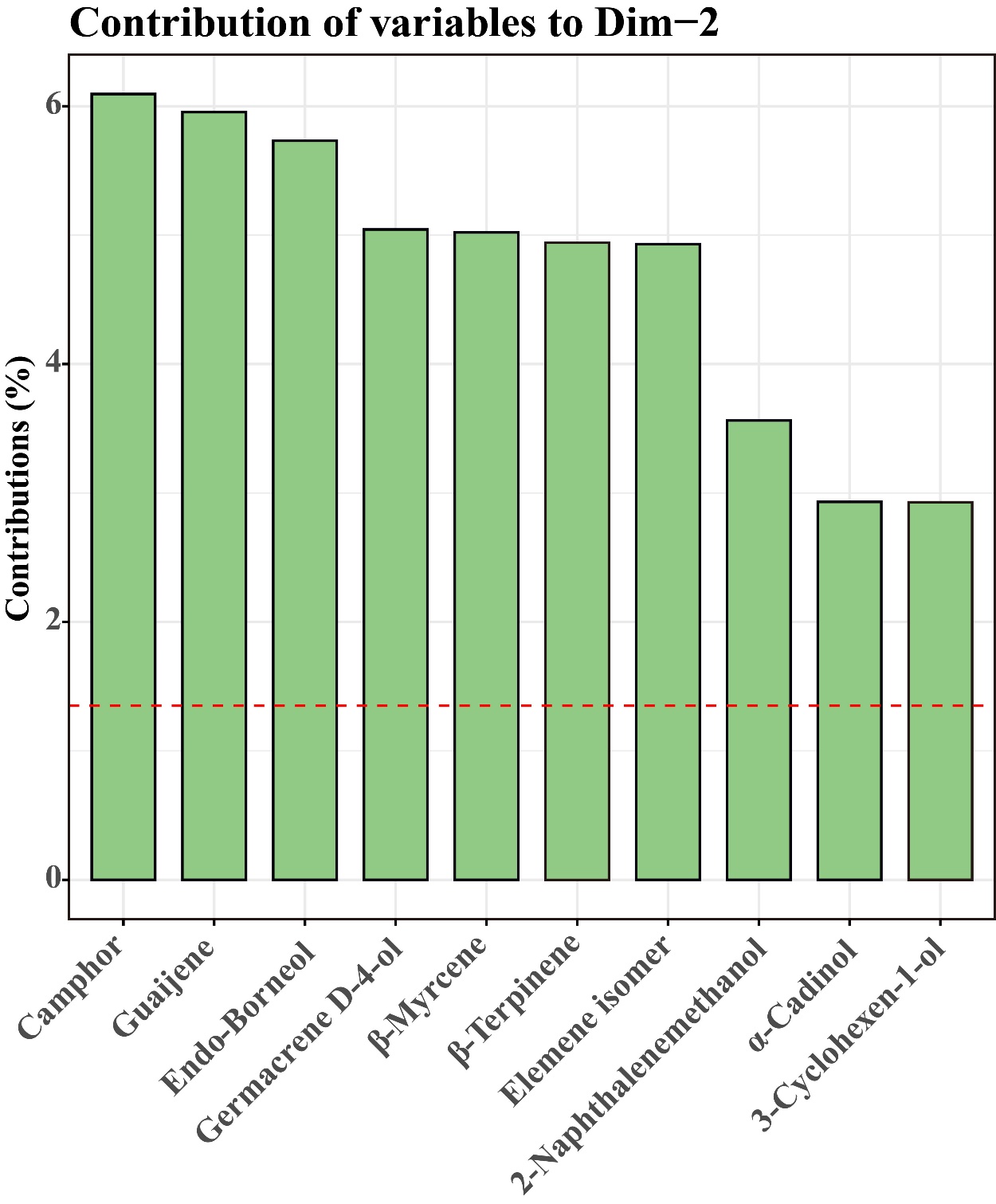


Appendix 7. The variables selected and their contribution to PC2.


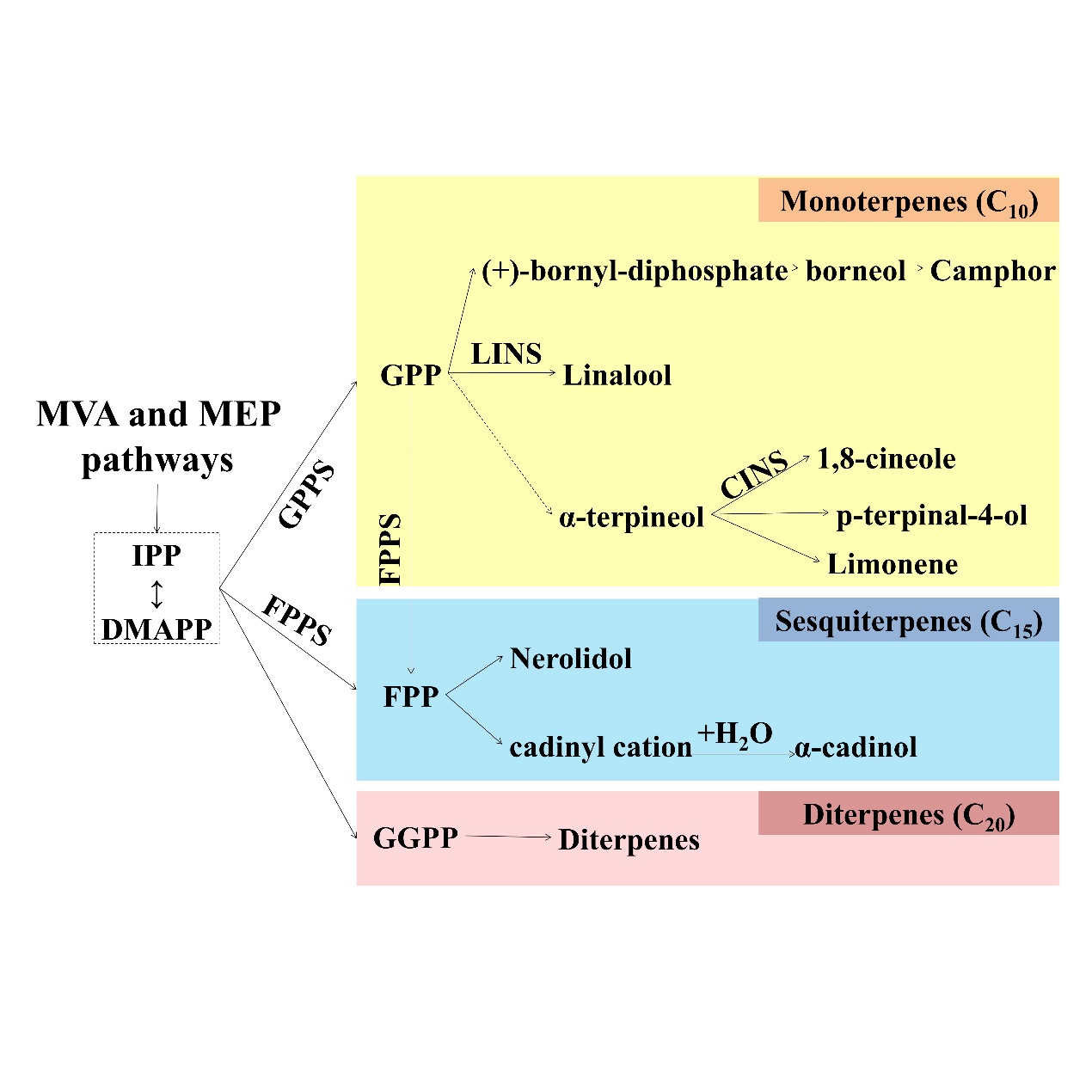


Appendix 8. The biosynthetic pathways of major compounds found in the leaf essential oil from three *Cinnamomum* species. MEP pathway: methyl-erythritol 4-phosphate pathway; MVA pathway: mevalonate pathway; IPP: isopentenyl diphosphate; DMAPP: dimethylallyl diphosphate; GPP: geranyl diphosphate; FPP: farnesyl diphosphate; GPPS: GPP synthase; FPPS: FPP synthase; LINS: linalool synthase; CINS: cineole synthase; GGPP: Geranylgeranyl pyrophosphate.
